# New hope for Nutlin-3a therapy for pulmonary arterial hypertension

**DOI:** 10.3389/fphar.2013.00087

**Published:** 2013-07-10

**Authors:** Jutaro Fukumoto, Richard Lockey, Narasaiah Kolliputi

**Affiliations:** Division of Allergy and Immunology, Department of Internal Medicine, Morsani College of Medicine, University of South FloridaTampa, FL, USA

Pulmonary Arterial Hypertension (PH) is a chronic, progressive, and fatal lung disease characterized by narrowing of pulmonary arteries associated with muscular thickening and intimal proliferation, known as remodeling. If untreated, the 5-year survival rate of PH is 34% (McLaughlin et al., 2002). Unfortunately, there is no systematic strategy established to effectively manage this devastating disease. Currently, the best curative option for PH is lung transplantation. It, however, is applicable for only a few patients mainly due to the lack of suitable donors. Moreover, even if lung transplantation is successfully performed, the patients have to take immunosuppressant for the rest of their life. Administration of long-term immunosuppressant has been associated with high incidence of side effects including severe and sometimes fatal infections.

The FDA-approved medications for PH fall into three categories: (i) prostacyclin derivative, which is a very potent vasodilator and also a inhibitor of pulmonary artery smooth muscle cell (PASMC) proliferation, (ii) endothelin-1 receptor antagonist, which counteracts vasoconstriction and PASMC proliferation, and (iii) phosphodiesterase-5 inhibitor, which causes pulmonary vasodilatation and inhibition of PASMC proliferation. These vasodilators have certainly achieved significant advances in the management of PH during the last decade. However, because of their short half-lifes and narrow therapeutic ranges, close monitoring and/or follow-up is required to avoid excessive side effects involved in vasodilation. Some PH patients with severe conditions even require continuous intravenous or subcutaneous administration of these drugs, which can be a big clinical challenge for both patients and health care providers. To get a better way in managing PH conditions and in maintaining long-term patient adherence, a new drug that specifically and strongly blocks PASMC proliferation, a key pathological event in PH, is desired.

In the last decade, a good line of evidence has revealed that there is a variety of similarities existent between PH and cancer (McMurtry et al., 2005; Dromparis et al., 2010; Aljubran et al., 2012). Two representatives of them are uncontrolled cellular proliferation and resistance to apoptosis. In the recent issue of *Circulation, Mouraret et al.* present an elegant study on the effects of Nutlin-3a on PH (Mouraret et al., 2013). Nutlin-3a, which is a cis-imidazoline analog, was originally developed as an anti-cancer drug. It stabilizes p53 by inhibiting the binding of MDM2 (murine double minute 2), a p53-specific E3 ubiquitin ligase that promotes p53 degradation (Vassilev et al., 2004). p53, a representative tumor suppressor protein, plays a critical role in tumor suppression through several different mechanisms primarily involving the DNA repair process. Recently, it has been reported that p53 deletion deteriorates hypoxia-induced PH in mice (Mizuno et al., 2011). Consistent with this report, *Mouraret et al.* revealed that a p53 stabilizer Nutlin-3a reversed PH in three distinct experimental mouse models with only limited side effects noted in control mice without PH. Moreover, they confirmed that Nutlin-3a requires p53 and p21 expression to work as an anti-PH drug showing that Nutlin-3a exerts no effects on hypoxia-exposed p53^−/−^ and p21^−/−^ mice. Intriguingly, the paper also shows that Nutlin-3a treatment of cultured human PASMCs results in cell growth arrest but not apoptosis, which is consistent with other reports using fibroblasts from mice and humans (Efeyan et al., 2007; Kumamoto et al., 2008). Therefore, it appears that Nutlin-3a has a totally different mechanism of action from currently available drugs and that it is not directly associated with relaxation of smooth muscle and vasodilation of the pulmonary arteries, which lessens the concern about the side effects related to vasodilation. If Nutlin-3a works well for treatment of PH patients and it becomes an approved therapeutic application, PH patients would be able to have more therapeutic options including combination therapies, which ultimately would lead to decreased number of PH patients with advanced stages. The benefit of expanded therapeutic options could have an impact in decreasing the number of PH patients waiting for lung transplantation. On the other hand, one minor drawback of using Nutlin-3a as a potential PH treatment is its slow clinical effect on the patients with severe PH due to its lack of direct vasodilatory effect. However, in spite of the above mentioned disadvantage, this new drug still has an enormous therapeutic potential for treatment of PH. The day may come before long when Nutlin-3a will come out as a blessing for PH patients.
